# Meta-analysis of the efficacies of amiodarone and nifekalant in shock-resistant ventricular fibrillation and pulseless ventricular tachycardia

**DOI:** 10.1038/s41598-017-13073-0

**Published:** 2017-10-04

**Authors:** Shiho Sato, Yoshito Zamami, Toru Imai, Satoshi Tanaka, Toshihiro Koyama, Takahiro Niimura, Masayuki Chuma, Tadashi Koga, Kenshi Takechi, Yasuko Kurata, Yutaka Kondo, Yuki Izawa-Ishizawa, Toshiaki Sendo, Hironori Nakura, Keisuke Ishizawa

**Affiliations:** 1 Department of Emergency Pharmaceutical Science, Graduate School of Medicine, Dentistry and Pharmaceutical Sciences, Okayama University, 1-1-1 Tsushima-naka, Okayama, 700-8530 Japan; 2 Department of Clinical Pharmacology and Therapeutics, Institute of Biomedical Sciences, Tokushima University Graduate School, 3-18-15 Kuramoto, Tokushima, 770-8503 Japan; 3 Department of Pharmacy, Tokushima University Hospital, 2-50-1 kuramoto-cho, Tokushima, 770-8503 Japan; 4 Department of Pharmacy, Nihon University Itabashi Hospital, 30-1 Oyaguchi-Kami Machi, Itabashi-ku, Tokyo, 173-8610 Japan; 5 South Miyagi Medical Center, Pharmaceutical Department, 38-1 Aza-Nishi, Ogawara, Shibata-gun, Miyagi, 989-1253 Japan; 6 Department of Clinical Pharmacy, Graduate School of Medicine, Dentistry and Pharmaceutical Sciences, Okayama University, 2-5-1 Shikata-cho, Kita-ku Okayama, 700-8558 Japan; 7 Drug Safety Research Laboratories, Shin Nippon Biomedical Laboratories, Ltd, 2438 Miyanoura, Kagoshima, 891-1394 Japan; 8 Department of Pharmacy, Okayama University Hospital, 2-5-1 Shikata-cho, Kita-ku, Okayama, 700-8558 Japan; 9 Department of Surgery, Beth Israel Deaconess Medical Center, Harvard Medical School, 330, Brookline Avenue, Boston, MA 02215 USA; 10 Department of Pharmacology, Institute of Biomedical Sciences, Tokushima University Graduate School, 3-18-15 Kuramoto, Tokushima, 770-8503 Japan

## Abstract

Amiodarone (AMD) and nifekalant (NIF) are used in the treatment of ventricular fibrillation or tachycardia; however, only few studies have been conducted on their efficacies. Therefore, a meta-analysis was conducted. Relevant sources were identified from PubMed, Cochrane Central Register of Controlled Trials, and Igaku Chuo Zasshi. The outcomes were short-term and long-term survival in patients with shock-resistant ventricular fibrillation /pulseless ventricular tachycardia. Thirty-three studies were analysed. The results showed that, compared to the control treatment, AMD did not improve short-term survival (odds ratio (OR): 1.25, 95% confidence interval (CI): 0.91–1.71) or long-term survival (OR: 1.00, 95% CI: 0.63–1.57). However, compared to the control treatment, NIF significantly improved short-term survival (OR: 3.23, 95% CI: 2.21–4.72) and long-term survival (OR: 1.88, 95% CI: 1.36–2.59). No significant difference was observed in short-term survival (OR: 0.85, 95% CI: 0.63–1.15) or long-term survival (OR: 1.25, 95% CI: 0.67–2.31) between AMD- and NIF-treated patients. The results suggest that NIF is beneficial for short-term and long-term survival in shock-resistant ventricular fibrillation/pulseless ventricular tachycardia; however, the efficacy of AMD in either outcome is not clear.

## Introduction

The incidence of sudden cardiac arrest (SCA) is high. Moreover, SCA patients have a poor prognosis for survival. Out-of-hospital cardiac arrest (OHCA) that results in transportation in an ambulance to the hospital occurs in approximately 120,000 people per year^1^. In addition, the incidence of ventricular fibrillation (VF) in patients who have suffered OHCA is about 60% in Japan^2^. The current resuscitation guidelines recommend the use of amiodarone (AMD) for the treatment of shock-resistant adult VF/pulseless ventricular tachycardia (pVT) during cardiopulmonary resuscitation (CPR). However, lidocaine or nifekalant (NIF, approved for use only in Japan) may be administered if AMD is unavailable^3–5^. AMD and NIF are type III antiarrhythmic drugs. Randomized controlled trials (RCTs) and observational studies have shown that AMD and NIF cause better rates of survival until hospital admission than placebo or lidocaine do in VF/pVT patients^6–8^. Only a few RCTs and observational or retrospective studies have been conducted on the efficacies of AMD and NIF in the management of SCA. One of the reasons for this is that SCA occurs unexpectedly; therefore, large-scale clinical trials on it are difficult to conduct. In addition, there is a possibility that differences in treatment cannot be detected due to power shortage in a single research. In such a case, meta-analysis is a useful method of analysis because it integrates and evaluates multiple studies.

The aim of this study was to clarify the effects of AMD or NIF on the survival outcome of patients with shock-resistant VF/pVT. We conducted a meta-analysis of studies that compared the efficacies of the following: AMD versus control treatment (lidocaine, placebo, or non-treatment antiarrhythmic drugs), NIF versus control treatment (lidocaine, placebo, or non-treatment antiarrhythmic drugs), and AMD versus NIF.

## Results

**Figure 1:**
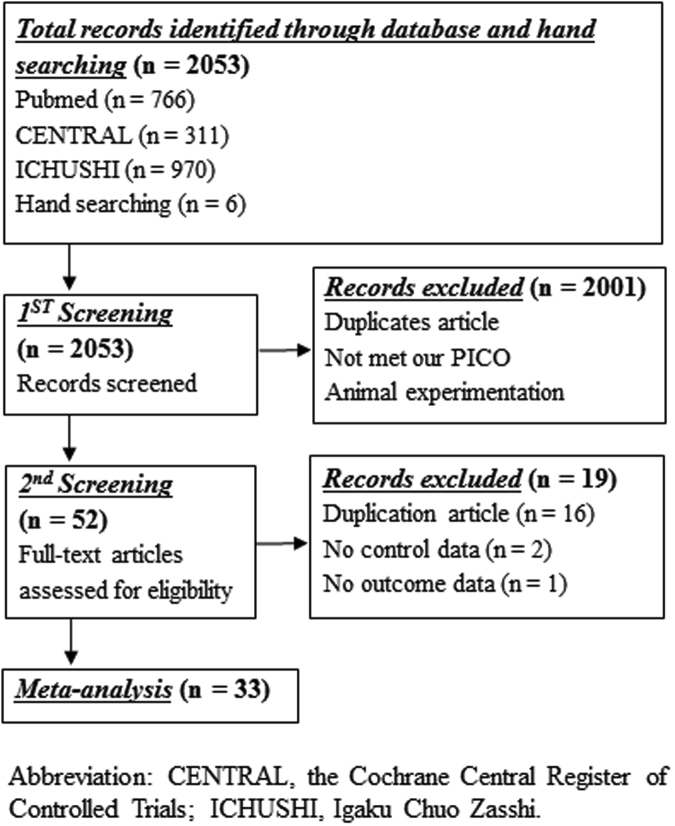
Literature screening process and results.

A total of 2053 studies were initially retrieved from the databases. After reviewing titles and abstracts, 2001 studies were excluded from the analysis. Therefore, the full texts of 52 reports were evaluated; however, 19 studies were further excluded. Finally, 33 studies^6–36^ were used in the qualitative synthesis and meta-analysis. The literature screening process and results are depicted in Fig. 1. The general characteristics of the included studies are shown in Supplementary Table S1. Seven of the included studies were RCTs^6,7,9,18,22,28,30^, 6 were observational studies^8,11,19,21,25^, and 20 were retrospective studies^10,12–17,19,20,23,24,26,27,29,31–36^.

### Methodological quality of studies

Assessment of methodological quality revealed that seven studies were RCTs (Supplementary Table S2). Of these, three had a low risk of bias, three had a high risk of bias, and one had an unclear risk of bias. Twenty-six studies were non-RCTs (Supplementary Table S3). Of these, two had a low risk of bias, 12 had a high risk of bias, and 12 had an unclear risk of bias.

### Meta-analysis and assessment of publication bias, AMD versus control treatment

**Figure 2:**
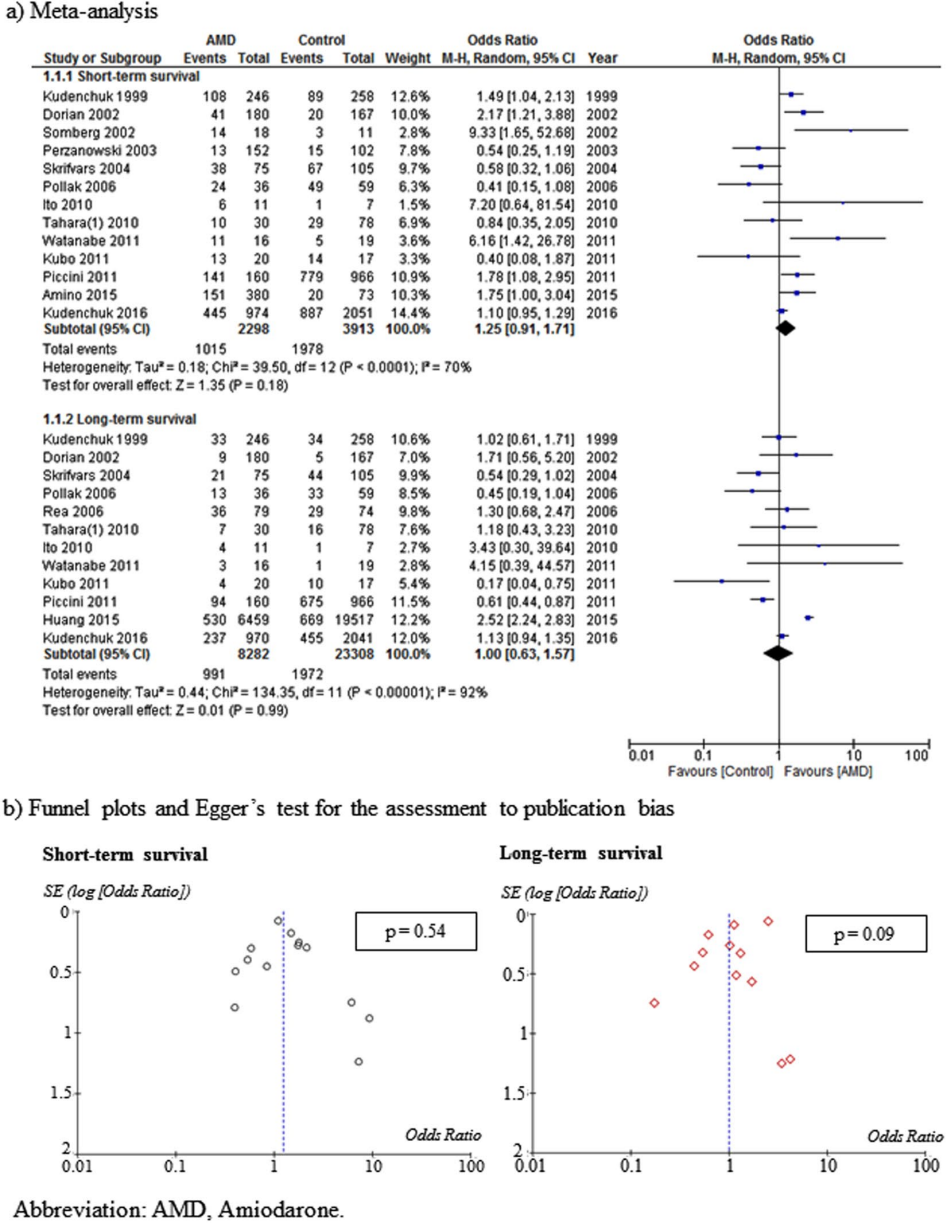
Meta-analysis and publication bias assessment for the effects of AMD on short-term survival and long-term survival compared with control treatment.

Fifteen studies (RCTs, 4; non-RCTs, 11) compared the effects of AMD (8831 patients) with those of a control treatment (23510 patients) during CPR. Furthermore, the risk of bias was low, high, and unclear in 5, 6, and 4 studies, respectively. Compared to the control group, AMD-treated patients did not show improved short-term survival (OR: 1.25, 95% CI: 0.91–1.71) or long-term survival (OR: 1.00, 95% CI: 0.63–1.57). Moreover, both short-term survival (*I* ^2^ = 70%) and long-term survival (*I* ^2^ = 92%) showed high heterogeneity when the AMD-treated and control groups were compared (Fig. 2a). Funnel plots and Egger’s tests indicated no possibility of publication bias in short-term survival (p = 0.54); however, there was possible publication bias in long-term survival (p = 0.09) (Fig. 2b).

### Meta-analysis and assessment of publication bias, NIF versus control treatment

**Figure 3:**
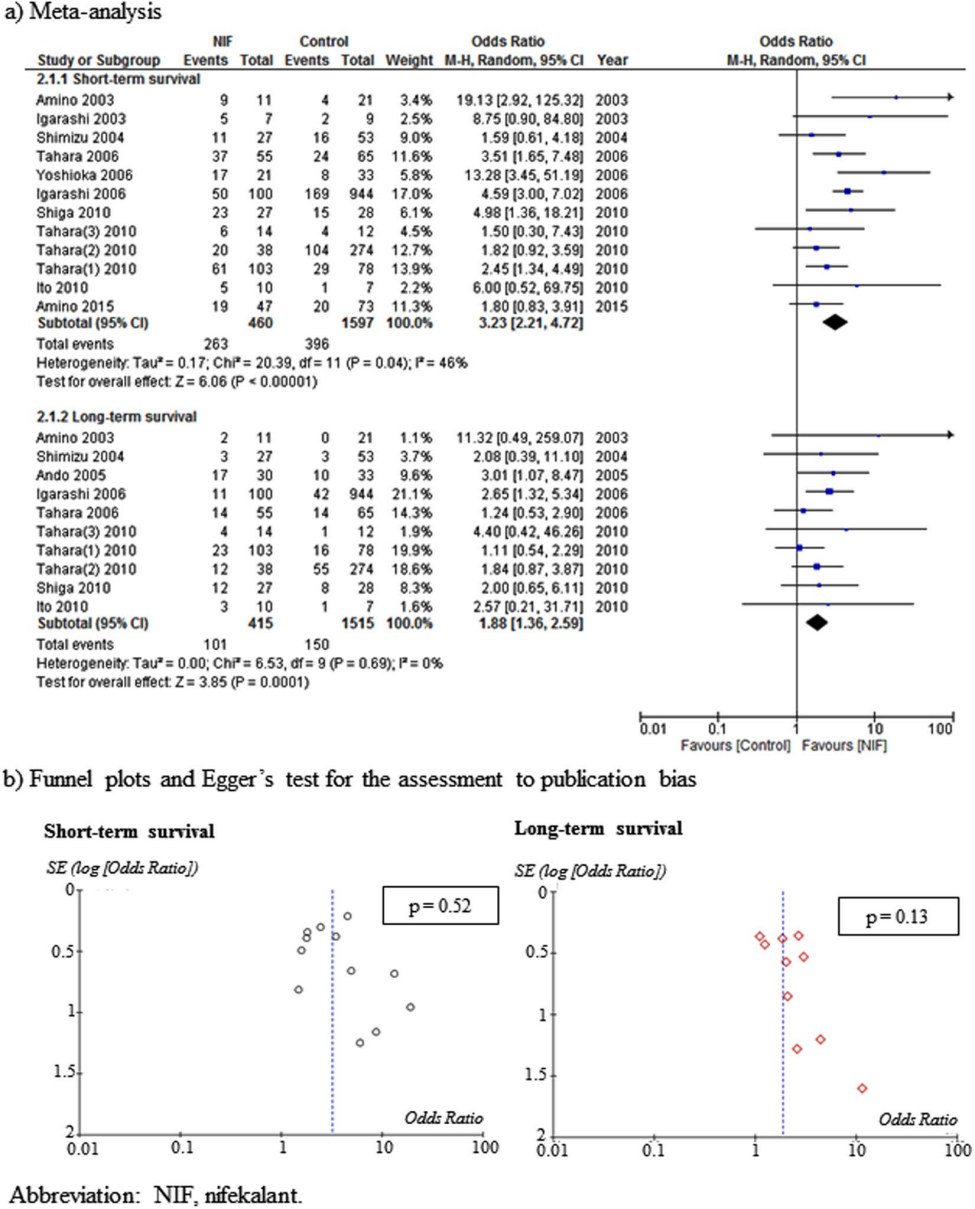
Meta-analysis and publication bias assessment for the effects of NIF on short-term survival and long-term survival compared with control treatment.

Thirteen studies (RCTs, 2; non-RCTs, 11) compared the effects of NIF (490 patients) with those of a control treatment (1630 patients) during CPR. The risks of bias of the included studies were as follows: low, 1; high, 8; and unclear, 4. NIF significantly improved short-term survival (OR: 3.23, 95% CI: 2.21–4.72) and long-term survival (OR: 1.88, 95% CI: 1.36–2.59) (Fig. 3a). Furthermore, heterogeneity was low in short-term survival (*I* ^2^ = 46%) and long-term survival (*I* ^2^ = 0%) when the NIF-treated and control groups were compared. Funnel plots and Egger’s tests indicated no possibility of publication bias in short-term survival (p = 0.52) or long-term survival (p = 0.13) (Fig. 3b).

### Meta-analysis and assessment of publication bias, AMD versus NIF

**Figure 4:**
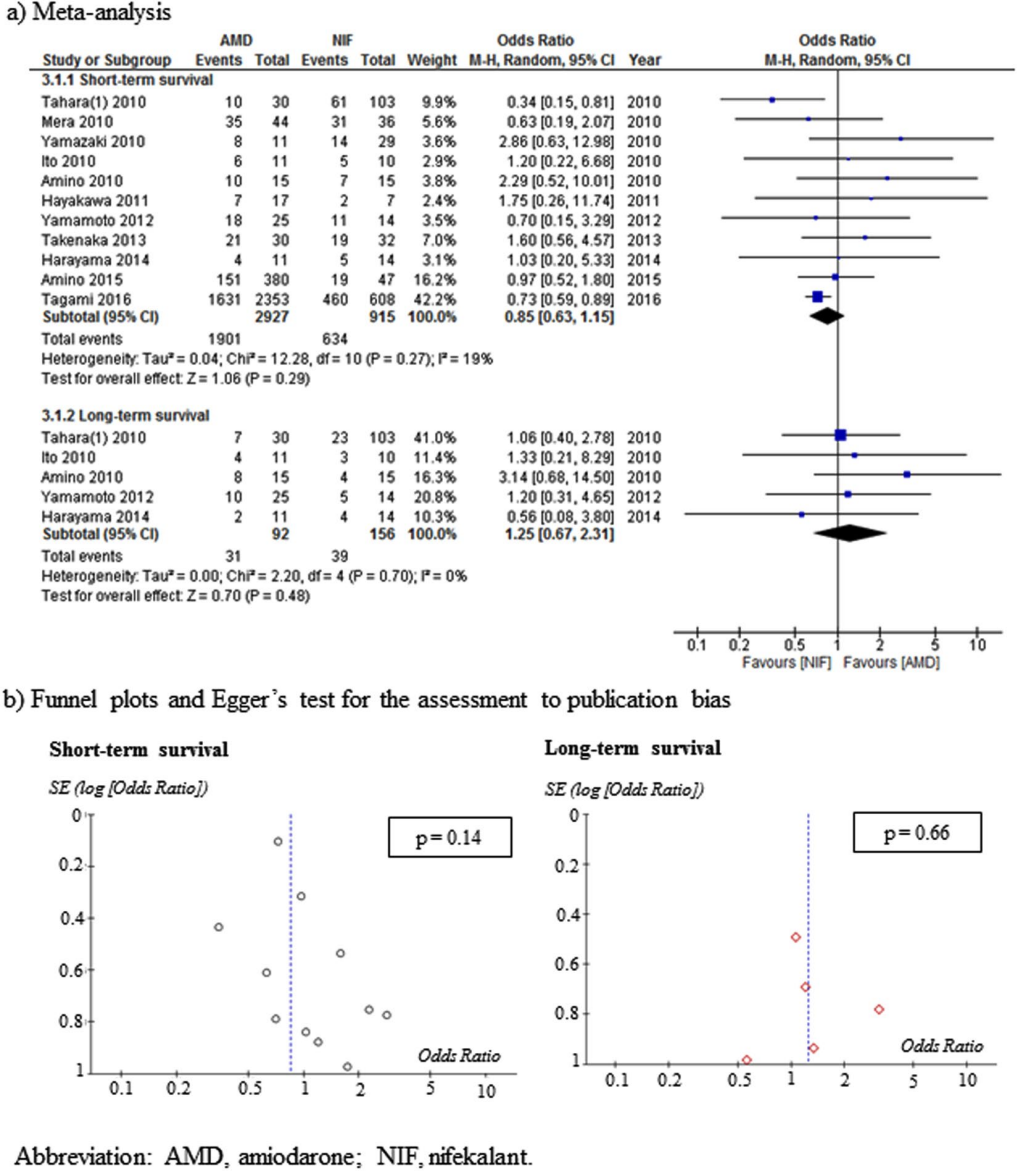
Meta-analysis and publication bias assessment for the effects of AMD on short-term survival and long-term survival compared with NIF.

Eleven studies (RCT, 1; non-RCTs, 10) compared the effects of AMD (2927 patients) with those of NIF (915 patients) during CPR. The risk of bias was low, high, and unclear in 1, 3, and 7 studies, respectively. No significant difference was found in short-term survival (OR: 0.85, 95% CI: 0.63–1.15) or long-term survival (OR: 1.25, 95% CI: 0.67–2.31) (Fig. 4a) when the AMD- and NIF-treated groups were compared. Furthermore, no significant heterogeneity was observed in short-term survival (*I* ^2^ = 19%) or long-term survival (*I* ^2^ = 0%). Funnel plots and Egger’s tests indicated no possibility of publication bias in short-term survival (p = 0.14) or long-term survival (p = 0.66) (Fig. 4b).

### Subgroup analyses

Subgroup analyses were performed to evaluate the effects of the various treatments in the different type of studies (Table 1). Differences in the effects of AMD and the control treatments were observed between the RCT (OR: 1.61, 95% CI: 1.03–2.49) and non-RCT (OR: 1.04, 95% CI: 0.61–1.77) studies with regard to short-term survival; however, this was not the case for long-term survival (RCTs, OR: 1.13, 95% CI: 0.95–1.33; non-RCTs, OR: 0.93, 95% CI: 0.46–1.87). Furthermore, when the NIF-treated and control groups were compared, the results obtained for the RCT and non-RCT studies were not different with regard to short-term survival (RCTs, OR: 15.03, 95% CI: 5.02–44.99; non-RCTs, OR: 2.80, 95% CI: 2.04–3.86)., but different with regard to long-term survival (RCTs, OR: 11.32, 95% CI: 0.49–259.07; non-RCTs, OR: 1.84, 95% CI: 1.33–2.54). Conversely, when the effects of AMD and NIF were compared, no different results were obtained between the RCT and non-RCT studies with regard to short-term survival (RCTs, OR: 2.29, 95% CI: 0.52–10.01; non-RCTs, OR: 0.80, 95% CI: 0.61–1.05) or long-term survival (RCTs, OR: 3.14, 95% CI: 0.68–14.50; non-RCTs, OR: 1.04, 95% CI: 0.53–2.05).

**Table 1:** Subgroup analysis in three comparison (RCTs or non-RCTs).

Comparison	Survival outcome	Study design	Odds ratio (95% CI)	Number of study
AMD vs. Control	Short-term	RCT	1.61 (1.03–2.49)*	4
		non-RCT	1.04 (0.61–1.77)	9
	Long-term	RCT	1.13 (0.95–1.33)	3
		non-RCT	0.93 (0.46–1.87)	9
NIF vs. Control	Short-term	RCT	15.03 (5.02–44.99)*	2
		non-RCT	2.80 (2.04–3.86)*	10
	Long-term	RCT	11.32 (0.49–259.07)	1
		non-RCT	1.84 (1.33–2.54)*	9
AMD vs. NIF	Short-term	RCT	2.29 (0.52–10.01)	1
		non-RCT	0.80 (0.61–1.05)	10
	Long-term	RCT	3.14 (0.68–14.50)	1
		non-RCT	1.04 (0.53–2.05)	4

Abbreviation: AMD, amiodarone; NIF, nifekalant; RCT, randomized controlled trial; CI, confidence interval. *p < 0.05.

### Sensitivity analysis

Only studies that had a low risk of bias were analysed. When the AMD-treated and control groups were compared, it was observed that AMD significantly improved short-term survival (OR: 1.50, 95% CI: 1.14–1.98, n = 5) but not long-term survival (OR: 0.95, 95% CI: 0.65–1.39, n = 4). Furthermore, when the NIF-treated and control groups were compared, it was observed that NIF did not improve short-term survival (OR: 1.80, 95% CI: 0.83–3.91, n = 1); however, no deductions could be made concerning long-term survival as it was not evaluated. Lastly, when the AMD- and NIF-treated groups were compared, no significant difference was found in the results for short-term survival (OR: 0.97, 95% CI: 0.52–1.80; n = 1). However, long-term survival was not evaluated in the study with low risk of bias.

## Discussion

AMD and NIF are class III antiarrhythmic drugs; however, their pharmacological profiles are different. The effects of AMD include blocking Na^+^ channels, Ca^2+^ channels, and K^+^ channels. It is also a β-adrenergic receptor blocker. In contrast, NIF is a pure K^+^ channel blocker. In this study, we evaluated the effects of AMD or NIF on short-term and long-term survival in adult patients with shock-resistant VF/pVT in a meta-analysis. Majority of the studies analysed were non-RCTs (79%). All the studies were conducted within a 20-year period (1994–2015) in various countries including the United States of America, Canada, Hungary, Finland, Taiwan, and Japan. The guidelines used for CPR varied among the studies.

Among the major RCTs studied^6,7^, AMD was reported to improve short-term survival but not long-term survival in out-of-hospital VF patients. AMD is recommended in the 2015 American Heart Association (AHA) Guidelines Update for CPR and Emergency Cardiovascular Care and Japan Resuscitation Council Guidelines 2015 (JRC Guideline 2015) as the first-line drug for treating shock-resistant VF/pVT patients during ACLS^4,5^. However, we could not confirm the effectiveness of AMD for long-term or short-term survival in this meta-analysis. In the subgroup analysis, different results were obtained for the RCT and non-RCT studies. AMD improved short-term survival in the RCTs but not in the non-RCTs. Furthermore, the sensitivity analysis showed that, the improved short-term survival occurred only in the studies with low risk of bias. We considered the following factors as reasons for these results. 1) It was observed that many of the non-RCT reports indicated statistically significant differences in patient background factors, which are considered as confounding factors, between the AMD-treated and control groups. The confounding factors included defibrillation frequency, epinephrine dosage, patient age, and time until drug administration, among others. There was no study that aligned the patient background between AMD treatment group and control group by using such as propensity score. These confounding factors may have influenced the efficacy assessment of AMD and affected survival outcome in the RCTs and non-RCTs. 2) Regarding the ability to improve survival until hospital admission, AMD was found to be superior to placebo treatment, but not to lidocaine^18^. In the present analysis, placebo treatment, lidocaine, and non-treatment antiarrhythmic drugs were termed control group. The results indicate that AMD is more effective than placebo treatment is in treating shock-resistant VF/pVT; however, it appears that AMD and lidocaine have a similar efficacy. 3) In the 2015 AHA and JRC guidelines, the recommended dose of AMD for shock-resistant VF/VT patients is 300 mg as a bolus; however, this usage is not approved in some countries. Various dosages of AMD, such as 125 mg over 5–10 min and 150–300 mg over a few seconds to 1 min, were used until 2013, when the resuscitation dose (300-mg bolus injection) was approved in Japan. AMD has hemodynamic side effects such as bradycardia and blood pressure reduction, which are due to its multichannel blocking action. However, the side effects can be avoided by slowing the infusion rate^37^. Furthermore, it has been reported that, survival rate until hospital admission in Japan is higher in patients who receive 150 mg AMD than in those who receive 300 mg AMD^21^. These indicate that differences in dosage and infusion rate may influence the effects of AMD. 4) Polysorbate 80 is a surfactant that has been reported to lower blood pressure in dogs^38^. The effects of AMD alone^9,18^ or polysorbate 80 as a control treatment^6,7^ have been studied in RCTs. However, the effects of polysorbate 80 have not been excluded in non-RCTs^10–17,19–21^. It is therefore possible that polysorbate 80 affects the efficacy of AMD. In this meta-analysis, it was not possible to clarify the effects of AMD on short-term survival as reported in the RCTs. 5) From the analysis, fewer non-RCTs had low risk of bias than the RCTs had (non-RCTs, 18%; RCTs, 75%). Therefore, it is possible that some bias (such as that from confounding factors) was introduced into the efficacy evaluation of AMD in the non-RCTs.

Among small RCT^22,28^, NIF was reported to improve short-term survival, not long-term survival in out-of-hospital VF patients. NIF is recommended in the JRC Guidelines 2015 as the second-line drug for treating shock-resistant VF/pVT patients during ACLS. We were able to confirm the effects of NIF on short-term and long-term survival in this meta-analysis. There were similar results when the RCTs and non-RCTs were compared. In the sensitivity analysis, there was only one study that had a low risk of bias, and no significant effects of NIF on short-term survival were observed. It has been reported that NIF reduces defibrillation threshold. In addition, the time to achieve defibrillation success is shorter with NIF than it is with AMD^30,35^. Furthermore, NIF is a pure potassium channel blocker; therefore, its hemodynamic side effects are probably of a lesser severity than those of AMD are. The above-mentioned facts suggest that NIF is a very useful agent for resuscitation. Moreover, the findings of the present analysis suggest that NIF is effective for both short-term and long-term survival. However, because there was only one study that had a low risk of bias (non-RCTs, 9%; RCT, 0%), it is possible that some bias was introduced into the efficacy evaluation of NIF.

Among small-RCT^30^, AMD and NIF are reported to have no differences in their effects on short-term and long-term survival in out-of-hospital VF patients. In the present analysis, there was no statistically significant difference in short-term or long-term survival between the AMD- and NIF-treated patients or between the RCT and non-RCT studies. The results of an experiment conducted in pigs showed that NIF caused a higher survival rate than AMD does^39^. However, another study using pigs^40^ and some clinical trials^19–21,29–36^ have indicated that AMD and NIF have a similar efficacy. All the studies evaluated in this meta-analysis were conducted at advanced medical facilities in Japan (special functioning hospitals). In addition, there have been many studies conducted in Japan in which different dosages of the drugs (e.g., AMD, 125 mg over 5 min or 150 mg over 1 min; NIF, 0.15 mg/kg/min) have been evaluated. The above-mentioned factors may have had some impact on comparing the efficacy of AMD and NIF. The results of this meta-analysis suggest that NIF and AMD have a similar efficacy. However, since the number of studies analysed was few and several of them were of low quality (low risk of bias: RCT, 0%; non-RCTs, 10%), there may have biases in the efficacy evaluation of the drugs.

The possibility of publication bias was considered low in the three comparisons (AMD versus control treatment, NIF versus control treatment, and AMD versus NIF).

In order to clarify the efficacies of AMD and NIF during ACLS, large-scale studies such as multicentre collaborative researches must be conducted and the use of several databases will be required. In addition, it is essential that patient characteristics are similar between groups, especially in non-RCTs, to allow for effective comparisons to be made. This analysis had some limitations. Firstly, several of the reports analysed were non-RCTs and conference articles. In addition, the studies analysed were conducted during different periods and in different countries. Moreover, different CPR guidelines were used in the various studies. Lastly, patients who were concomitantly administered antiarrhythmic drugs were not excluded from the analysis.

## Conclusion

NIF may be effective for short-term and long-term survival in shock-resistant VF/pVT patients. However, the effects of AMD on either outcome could not be clarified.

## Methods

We conducted the meta-analysis according to the Preferred Reporting Items for Systematic Reviews and Meta-Analyses (PRISMA) guidelines^41^. The PRISMA checklist is shown in Supplementary Table S4.

### Eligibility criteria

We identified studies according to the following PICO criteria: Patients (adult patients who had suffered out/in-hospital cardiopulmonary arrest (CPA) and had VF or pVT were recruited for the studies), **I**ntervention (AMD or NIF was administered during advanced cardiovascular life support (ACLS)), **C**omparison (as control) (lidocaine, placebo, or a non-treatment antiarrhythmic drug was administered during ACLS), and **O**utcome (the reporting outcomes were short-term survival (defibrillation success, VF/pVT termination, return to spontaneous circulation, survival until admission to the hospital/intensive care unit, and three-hour survival) and long-term survival (30-day survival, 1-year survival, and survival until discharge from hospital)).

### Study selection and data extraction

To identify studies that were relevant to the analysis, we searched the PubMed, Cochrane Central Register of Controlled Trials (CENTRAL), and Igaku Chuo Zasshi databases for articles published until December 2016 using the following keywords: “amiodarone”, “nifekalant”, “ventricular fibrillation”, “pulseless ventricular tachycardia”, and “cardiopulmonary arrest”. The search formula is shown in Supplementary Table S5. We also manually searched through the reference lists of retrieved articles to trace other relevant studies. The search was not limited to articles published in a particular language. Two authors (S.S. & S.T.) independently screened the titles and abstracts of all selected articles (first screening). We then assessed the full text of each article for eligibility, which constituted the second screening. Articles that did not meet our PICO criteria, such as review articles, paediatric reports, results of animal experimentation, case reports, reports on pharmacokinetics, and duplicate reports, were excluded from the analysis during the screening. Disagreements about which studies to exclude from the analysis were resolved based on discussions between two authors (S.S. & S.T.). The following data were extracted from the included studies: first author’s last name, publication year, study design, settings of the study, patients’ characteristics, rhythms of arrest, details of sample collection, treatment regimens, and outcomes reported. Inconsistencies in data extraction were resolved through discussion. In addition, authors were contacted for clarifications when needed.

### Evaluation of the methodological quality of included studies

We assessed the methodological quality of each included RCT by using the risk of bias tool recommended by the Cochrane Collaboration (London, UK)^42^. In addition, the studies were evaluated using the following quality domains: random sequence generation, allocation concealment, blinding of participants as well as personal and outcome assessors, incomplete outcome data, selective outcome reporting, and other potential threats to validity. Non-RCTs (such as observational and retrospective studies) were evaluated using the Risk of Bias Assessment Tool for Non-randomized Studies^43^. This was followed by assessment using the following quality domains: selection of participants, confounding variables, measurement of exposure, blinding of outcome assessments, incomplete outcome data, and selective outcome reporting. The assessment was performed independently by two authors (S.S. & S.T.). A study was considered as having a low risk of bias if each key domain was found to have a low risk of bias. Alternatively, a study was considered as having high or unclear risk of bias if one or more key domains were found to have high or unclear risk of bias.

### Comparison groups

The effects of the following were compared: AMD versus control treatment (lidocaine, placebo, or non-treatment antiarrhythmic drugs), NIF versus control treatment (lidocaine, placebo, or non-treatment antiarrhythmic drugs), and AMD versus NIF.

### Meta-analysis

Meta-analysis was performed using RevMan5.1^®^ software (The Cochrane Collaboration). Outcomes were determined using a random-effects model and taking into consideration the variability of CPR practice, the different countries in which the studies were conducted, the different comorbidities of patients, and the different treatment strategies, among other factors. Outcomes were evaluated using odds ratio (OR) with 95% confidence interval (95% CI). Between-study heterogeneity was considered high if *I* ^2^ statistic was >50%.

### Assessment of publication bias

R metafor software (Software Foundation’s GNU General Public License) was used to assess the degree of publication bias both graphically and statistically using the funnel plot asymmetry test (Egger’s test)^44^. P values < 0.1 were considered statistically significant according to the asymmetry of the funnel plot (indicating the possibility of publication bias).

### Subgroup analysis

We performed subgroup analyses to evaluate RCTs and non-RCTs.

### Sensitivity analysis

We also performed a sensitivity analysis by excluding studies that had a high or unclear risk of bias.

## Electronic supplementary material

Supplementary Table

## References

[1] Fire and Disaster Management Agency. Current status of emergency rescue http://www.fdma.go.jp/neuter/topics/kyukyukyujo_genkyo/h28/01_kyukyu.pdf (2016).

[2] SOS-KANTO Committee. Incidence of ventricular fibrillation in patients with out-of-hospital cardiac arrest in Japan: survey of survivors after out-of-hospital cardiac arrest in Kanto area (SOS-KANTO). *Circ. J.* **69**, 1157 (2005). 10.1253/circj.69.1157 16195609

[3] Callaway, C.W. *et al*. Part 4: Advanced Life Support: 2015 International Consensus onCardiopulmonary Resuscitation and Emergency Cardiovascular Care Science WithTreatment Recommendations. *Circulation* **132**, suppl 1, S84–S145, 10.1161/CIR.0000000000000273. 10.1161/CIR.0000000000000273 26472860

[4] Kleinman ME (2015). Part 5: Adult Basic Life Support and CardiopulmonaryResuscitation Quality: 2015 American Heart Association Guidelines Update forCardiopulmonary Resuscitation and Emergency Cardiovascular Care. Circulation.

[5] Japan Resuscitation Council. Part 2: Adult Basic Life Support and Cardiopulmonary Resuscitation Quality. Japan Resuscitation Council Guideline 2015.http://www.japanresuscitationcouncil.org/wp-content/uploads/2016/04/0e5445d84c8c2a31aaa17db0a9c67b76.pdf (2015).

[6] Kudenchuk PJ (1999). Amiodarone for resuscitation after out-of-hospital cardiac arrest due to ventricular fibrillation. N Engl J Med..

[7] Dorian P (2002). Amiodarone as compared with lidocaine for shock-resistant ventricular fibrillation. N. Engl. J. Med..

[8] Shiga T (2010). Nifekalant versus lidocaine for in-hospital shock-resistant ventricularfibrillation of tachycardia. Resuscitation.

[9] Somberg JC (2002). Intravenous lidocaine versus intravenous amiodarone (in a newaqueous formulation) for incessant ventricular tachycardia. Am. J. Cardiol..

[10] Perzanowski C, Osur M, Myrin B, Sehra R (2003). Amiodarone does improve survivalin out of hospital cardiac arrest in a rural and semi-rural setting. Europace..

[11] Skrifvars MB (2004). The use of undiluted amiodarone in the management ofout-of-hospital cardiac arrest. Acta. Anaesthesiol. Scand..

[12] Pollak PT, Wee V, Al-Hazmi A, Martin J, Zarnke KB (2006). The use of amiodarone for in-hospital cardiac arrest. Can. J. Cardiol..

[13] Rea RS (2006). Comparing intravenous amiodarone or lidocaine, or both, outcomes for inpatients with pulseless ventricular arrhythmias. Crit. Care. Med..

[14] Piccini JP (2011). Antiarrhythmic drug therapy for sustained ventricular arrhythmias complicating acute myocardial infarction. Crit. Care Med..

[15] Watanabe E, Asai T, Minami K, Nakano H, Asaoka M (2011). Efficacy of intravenous amiodarone for out-of hospital refractory ventricular fibrillation. Prog. Med..

[16] Kubo S (2011). Present used status of intravenous amiodarone infusion for cardiopulmonary resuscitation. Prog. Med..

[17] Huang CH (2015). Amiodarone, lidocaine or neither for shockable cardiac arrest patients in emergency room – A nationwide cohort study. Resuscitation.

[18] Kudenchuk PJ (2016). Amiodarone. lidocaine, or placebo in out-of-hospital cardiac arrest. N. Engl. J. Med..

[19] Tahara Y (2010). Comparison of amiodarone and nifekalant for shock-resistant ventricular fibrillation: SOS-KANTO study. Cardioangiology.

[20] Ito H (2010). Defibrillation effects of intravenous amiodarone, nifekalant, and lidocaine in patients with out –of-hospital ventricular fibrillation. Shinzo.

[21] Amino M (2015). Nifekalant hydrochloride and amiodarone hydrochloride result in similar improvement for 24-hour survival in cardiopulmonary arrest patients: the SOS-KANTO 2012 study. J. Cardiovasc. Pharmacol..

[22] Amino M (2003). Efficacy of nifekalant hydrochloride for life-threatening ventricular tachyarrhythmias in patients with resistance to lidocaine: a study of patients with out-of-hospital cardiac arrest. J. Cardiol..

[23] Igarashi M (2003). Defibrillation effects of nifekalant in patients with out-of-hospital ventricular fibrillation. Shinzo.

[24] Shimizu K (2004). The effectiveness of nifekalant for out-of-hospital cardiopulmonary arrest with intractable ventricular fibrillation. J. Jpn. Soc. Intensive Care Med..

[25] Igarashi M (2006). Efficacy of administration of nifekalant in patients with out-of-hospital ventricular fibrillation from SOS-Kanto reports. JAAM..

[26] Ando, J., *et al*. Efficacy of nifekalant hydrochloride in the treatment of fatal ventricular arrhythmia in patients with ischemic heart disease. *Int. Heart J*. 647–656 (2005). 10.1536/ihj.46.647 16157956

[27] Tahara Y (2006). Comparison of nifekalant and lidocaine for the treatment of shock-refractory ventricular fibrillation. Circ. J..

[28] Yoshioka K (2006). Can nifekalant hydrochloride be used as a first-line drug for cardiopulmonary arrest (CPA)?- Comparative study of out-of-hospital CPA with acidosis and in-hospital CPA without acidosis-. Circ. J..

[29] Yamazaki T (2010). The effects of nifekalant and amiodarone on lethal ventricular arrhythmia. SHINZO.

[30] Amino M (2010). Comparative study of nifekalant versus amiodarone for shock-resistant ventricular fibrillation in out-of hospital cardiopulmonary arrest patients. J. Cardiovasc. Pharmacol..

[31] Mera H (2010). Clinical trial of ventricular tachycardia/ventricular fibrillation patients undergoing CCU management. J. Arrhythmia.

[32] Hayakawa K (2011). Investigation of intravenous of amiodarone for out-of-hospital cardiopulmonary arrest with witnesses. Prog. Med..

[33] Yamamoto M (2012). Comparative study of nifekalant versus amiodarone for out- of-hospital cardiopulmonary arrest patients. J. Arrhythmia..

[34] Takenaka K (2013). Comparison of amiodarone and nifekalant for the treatment of cardiogenic shock patients with ventricular fibrillation. Circ. J..

[35] Harayama N (2014). Comparison of nifekalant and amiodarone for resuscitati out-of-hospital cardiopulmonary arrest resulting from shock-resistant ventricular fibrillation. J. Anesth..

[36] Tagami T (2016). Amiodarone or nifekalant upon hospital arrival for refractory ventricular fibrillation after out-of-hospital cardiac arrest. Resuscitation.

[37] Marinelli A, Capucci A (2012). Amiodarone (Nexterone) injection for the treatment and prophylaxis of frequently recurring ventricular fibrillation. Expert. Opin. Pharmacother..

[38] Grough WB, Zeiler RH, Barreca P, EI-Sherif N (1982). Hypotensive action of commercial intravenous amiodarone and polysorbate 80 in dogs. J. Cardiovasc. Pharmacol..

[39] Karlis G (2015). Nifekalant versus amiodarone in the treatment of cardiac arrest: an experimental study in a swine model of prolonged ventricular fibrillation. Cardiovasc. Drugs Ther..

[40] Ji XF (2010). Comparison of the efficacy of nifekalant and amiodarone in a porcine model of cardiac arrest. Resuscitation.

[41] Liberati A (2009). The PRISMA statement for reporting systematic reviews and meta-analyses of studies that evaluate healthcare interventions: explanation and elaboration. BMJ..

[42] Higgins, J. P. T., Altman, D. G. & Sterne, J. A. C. Assessing risk of bias in included studies. Cochrane Handbook for systematic Reviews of Interventions Version 5.1.0 (updated http://www.cochrane-handbook.org (2011).

[43] Kim SY (2013). Testing a tool for assessing the risk of bias for nonrandomized studies showed moderate reliability and promising validity. J. Clin. Epidemiol..

[44] Egger M, Smith GD, Schneider M, Minder C (1997). Bias in meta-analysis detected by a simple, graphical test. BMJ..

