# Supplementary material for: Meta-analysis of the efficacies of amiodarone and nifekalant in shock-resistant ventricular fibrillation and pulseless ventricular tachycardia
Source: Sci Rep. 2017 Oct 4;7:12683. doi: 10.1038/s41598-017-13073-0 (PMC5627292; doi:10.1038/s41598-017-13073-0)
Supplement: Supplementary file 1 — Supplementary Table [file 41598_2017_13073_MOESM1_ESM.doc]

**Meta-analysis of the efficacies of amiodarone and nifekalant in shock-resistant ventricular fibrillation and pulseless ventricular tachycardia**

Shiho Sato^1, 4^, Yoshito Zamami^1, 2, 3*^, Toru Imai^4^, Satoshi Tanaka ^5^, Toshihiro Koyama^6^, Takahiro Niimura^2^, Masayuki Chuma^4^, Tadashi Koga^7^, Kenshi Takechi^3^, Yasuko Kurata^8^, Yutaka Kondo^9^, Yuki Izawa-Ishizawa^10^, Toshiaki Sendo^9^, Hironori Nakura^1^ & Keisuke Ishizawa^2, 3^

1. Department of Emergency Pharmaceutical Science, Graduate School of Medicine,

Dentistry and Pharmaceutical Sciences, Okayama University, 1-1-1 Tsushima-naka,

Okayama 700-8530, Japan.

2. Department of Clinical Pharmacology and Therapeutics, Institute of Biomedical Sciences, Tokushima University Graduate School, 3-18-15 Kuramoto, Tokushima, 770-8503, Japan.

3. Department of Pharmacy, Tokushima University Hospital, 2-50-1 kuramoto-cho, Tokushima, 770-8503 Japan.

4. Department of Pharmacy, Nihon University Itabashi Hospital, 30-1 Oyaguchi-Kami

Machi, Itabashi-ku, Tokyo, 173-8610, Japan.

5. South Miyagi Medical Center, Pharmaceutical Department, 38-1 Aza-Nishi, Ogawara, Shibata-gun, Miyagi 989-1253, Japan.

6. Department of Clinical Pharmacy, Graduate School of Medicine, Dentistry and

Pharmaceutical Sciences, Okayama University, 2-5-1 Shikata-cho, Kita-ku, Okayama,

700-8558, Japan.

7. Drug Safety Research Laboratories, Shin Nippon Biomedical Laboratories, Ltd, 2438 Miyanoura Kagoshima, 891-1394, Japan.

8. Department of Hospital Pharmacy, Okayama University Hospital, 2-5-1 Shikata-cho, Kita-ku, Okayama, 700-8558, Japan.

9. Department of Surgery, Beth Israel Deaconess Medical Center, Harvard Medical

School, 330, Brookline Avenue, Boston, MA 02215 USA

10. Department of Pharmacology, Institute of Biomedical Sciences, Tokushima University Graduate School, 3-18-15 Kuramoto, Tokushima, 770-8503 Japan.

Supplementary Table S1. Study characteristics

| First author, year of publication | Study design, period | Settings | Inclusion of patients | Intervention | Control | Outcomes |
| --- | --- | --- | --- | --- | --- | --- |
| **Kudenchuk, 1999^6^**  (ARREST) | RCT, DB,  1994-1997 | Two cities in the United States | OHCA, shock-resistant VF/pVT | **AMD** 300mg**,** n = 246 | **Placebo**(PS80),  n = 258 | SHA, SHD |
| **Dorian, 2002^7^**  (ALIVE) | RCT, DB,  1995-2001 | A multitiered out-of-hospital emergency response system in Canada | OHCA, shock-resistant VF | **AMD** 5mg/kg + placebo,  n = 180 | **LID** + placebo(PS80),  n = 167 | SHA, SHD |
| **Somberg, 2002^9^** | RCT, DB,  4-years | 63 centers from Canada, Hungary and United States | Shock-resistant VT | PS80 free **AMD** 150mg/ 2 min, n = 18 | **LID** 100mg/2 min,  n = 11 | VT termination |
| **Perzanowski,**  **2003^10^** | Retro,  1999-2002 | A large diverse geographic area in the United States | OHCA, shock-resistant VT/VF | **AMD**, n = 152 | **No AMD,** n = 102 | ROSC |
| **Skrifvars, 2004^11^** | Obs, US,  2000-2 | Helsinki EMS system in Finland | OHCA, shock-resistant VF/pVT | **AMD** 300mg, n = 75 | **No AMD**, n = 105 | SHA, SHD |
| **Pollak, 2006^12^** | Retro, 2years | Two centers in Canada | IHCA, VF/pVT | **AMD**, n = 36 | **No AMD**, n = 59 | ROSC, SHD |
| **Rea, 2006^13^** | Retro,  2000-2 | Three centers in the United States | IHCA, VF/pVT | **AMD**/bolus, n = 74 | **LID**, n = 79 | SHD |
| **Piccini, 2011^14^** | Retro,  1994-7 | GUSTO IIB and GUSTO III trials | IHCA, shock- resistant VF/VT | **AMD**(+ LID), n = 160 | **No AMD,** n = 966 | 3-hr survival, 25- day survival |
| **Kubo, 2011^16^** | Retro,  2007-10 | Nagoya City University Hospital in Japan | OHCA, shock-resistant VF/VT | **AMD** 300m/bolus or 125mg/10min, n = 20 | **No AMD**, n = 17 | ROSC, SHD |
| **Watanabe, 2011^15^** | Retro,  2007-9 | Okazaki Municipal Hospital in Japan | OHCA, refractory VF | **AMD** 125mg/10min,  n = 16 | **No AMD**, n = 19 | SHA, 30-day survival |
| **Huang, 2015^17^** | Retro,  2004-11 | The Taiwan National Health Insurance Research Database | OHCA, shock-resistant VF/pVT | **AMD,** n = 6459 | **No AMD,** n =19517 | 1-year survival |
| **Kudenchuk, 2016^18^**  (ROC-ALPS) | RCT, DB, 2012-5 | Ten cities in the United States | OHCA, shock-resistant VF/pVT | PS80 free **AMD** 300mg,  n = 974 | **LID**, n = 993 or  **Placebo**, n = 1059 | SHA, SHD |
| **Tahara(1), 2010^19^** | Retro,  1994-2009 | Yokohama City University Hospital in Japan | OHCA, shock-resistant VF | **AMD** 300mg, n = 30, or  **NIF** 0.3mg/kg, n = 103 | **LID**, n = 78 | SHA, SHD |
| **Ito, 2010^20^** | Retro,  2001-8 | Toho University in Japan | OHCA, shock-resistant VF | **AMD** 300mg/1 min, n = 11 or **NIF** 0.3mg/kg/1 min, n = 10 | **LID**, n = 7 | Defibrillation success, SHD |
| **Amino, 2015^21^**  (SOS-KANTO 2012) | Obs, US, 2012-3 | Sixty seven hospitals in Japan | OHCA, shock-resistant VF/VT | **AMD** 150-300mg,  n = 380 or **NIF**, n = 47 | **LID**, n = 73 | SHA |
| **Amino, 2003^22^** | RCT,  2000-1 | Tokai University Hospital in japan | OHCA, shock- resistant VF/VT | **NIF** 0.15-0.3mg/kg/5min, n = 11 | **LID or Procain-amide,** n = 21 | Defibrillation success, SHD |
| **Igarashi, 2003^23^** | Retro,  2001-2 | Toho University Hospital in japan | OHCA, shock- resistant VF | **NIF** 0.3mg/kg/1min,  n = 7 | **LID**, n = 9 | Defibrillation success, SHD |
| **Shimizu, 2004^24^** | Retro,  2000-2 | Saitama Red Cross Hospital in Japan | OHCA, shock-resistant VF | **NIF** 0.3mg/kg/5min,  n = 27 | **No NIF,** n = 53 | SHA, SHD |
| **Igarashi, 2006^25^**  (SOS-KANTO 2002) | Obs, US, 2002-3 | Fifty eight hospitals in Japan | OHCA, shock-resistant VF | **NIF**, n = 100 | **No NIF**, n = 944 | SHA, SHD |
| **Ando, 2005^26^** | Retro,  1996-2003 | Kokura Memorial Hospital in Japan | IHCA, shock-resistant VF/VT | **NIF**, n = 30 | **No NIF,** n = 33 | SHD |
| **Tahara, 2006^27^** | Retro,  1997-2004 | Yokohama City University Hospital in Japan | OHCA, shock-resistant VF | **NIF**, n = 55 | **LID**, n = 65 | SHA, SHD |
| **Yoshioka, 2006^28^** | RCT,  2000-3 | Tokai University Hospital in Japan | OHCA/IHCA, shock-resistant VF/pVT | **NIF** 0.15mg/kg/5min,  n = 21 | **LID** or **Procain-amide** or **Mg**, n = 33 | Defibrillation success |
| **Tahara(2), 2010^19^** | Obs, US, 2002-4 | Hospitals participating in SOS-KANTO 2002 study in Japan | OHCA, shock-resistant VF | **NIF**, n = 38 | **LID**, n = 274 | SHA, SHD |
| **Tahara(3), 2010^19^** | Obs, US,  2005 | Hospitals participating in SOS-KANTO VF/VT Study in Japan | OHCA, shock-resistant VF/VT | **NIF** 0.15mg/kg, n = 14 | **LID**, n = 12 | SHA, SHD |
| **Shiga, 2010^8^** | Obs,  2005-8 | Hospitals participating in RELIEF study in Japan | IHCA, shock-resistant VF/VT | **NIF**, n = 27 | **LID**, n = 28 | ROSC, SHD |
| **Yamazaki, 2010^29^** | Retro,  2003-9 | Kawasaki Saiwai Hospital in Japan | IHCA, shock-resistant VF/pVT | **AMD**, n = 11 | **NIF**, n = 29 | VF/pVT termination, 30 day-survival |
| **Amino, 2010^30^** | RCT,  2007-9 | Tokai University hospital in Japan | OHCA, shock-resistant VF | **AMD** 125mg/5min,  n = 15 | **NIF** 0.15mg/kg /5min,  n = 15 | SHA, SHD |
| **Mera, 2010^31^** | Retro,  2007-9 | Kyorin University Hospital in Japan | IHCA, shock-resistant VF/VT | **AMD,** n = 44 | **NIF**, n = 36 | VF/pVT termination |
| **Hayakawa, 2011^32^** | Retro,  2008-10 | Saitama Red Cross Hospital in Japan | OHCA, shock-resistant VF | **AMD** 150-300mg, n = 17 | **NIF**, n = 7 | ROSC |
| **Yamamoto, 2012^33^** | Retro,  2009-10 | Fujita Hearth University Hospital in Japan | OHCA, shock-resistant VF/VT | **AMD**, n = 25 | **NIF**, n = 14 | ROSC, SHD |
| **Takenaka, 2013^34^** | Retro,  2005-12 | Hirakatakousai Hospital in Japan | Shock-resistant VT | **AMD**, n = 30 | **NIF**, n = 32 | Defibrillation success |
| **Harayama, 2014^35^** | Retro,  2005-11 | Hospital of University of Occupational and Environmental Health in Japan | OHCA, shock-resistant VF | **AMD**/1min, n = 11 | **NIF**/1min, n = 14 | SHA, SHD |
| **Tagami, 2016^36^** | Retro,  2007-13 | The Japanese Diagnosis Procedure Combination inpatient database in Japan | OHCA, shock-resistant VF | **AMD,** n = 2353 | **NIF**, n = 608 | SHA |

Abbreviation: RCT, randomized controlled trials; DB, double-blind; Obs, observational studies; US, utstein-style; Retro, retrospective studies; OHCA, out-of-hospital cardiac arrest; IHCA, in-hospital cardiac arrest; VF, ventricular fibrillation; pVT, pulseless ventricular tachycardia; AMD, amiodarone; NIF, nifekalant; LID, lidocaine; PS80, polysorbate 80; Mg, magnesium; ROSC, return of spontaneous circulation; SHA, survival to hospital admission; SHD, survival to hospital discharge.

Supplementary Table S2. The risk of bias assessment for randomized controlled trials (Cochrane Risk of Bias Tool)

Abbreviation: AMD, amiodarone; NIF, nifekalant.

| Comparison | Random sequence generation | Allocation concealment | Blinding of participants, personal and outcome assessors | Incomplete outcome data addressed | Selective outcome reporting | Other potential to threats validity | Assessment of risk of bias across study |
| --- | --- | --- | --- | --- | --- | --- | --- |
|
| ***AMD vs. Control*** | | | | | | | |
| Kudenchuk, 1999 | Low | Low | Low | Low | Low | Low | Low |
| Dorian, 2002 | Low | Low | Low | Low | Low | Low | Low |
| Kudenchuk, 2016 | Low | Low | Low | Low | Low | Low | Low |
| Somberg, 2002 | Low | Low | Low | Unclear | Low | Low | Unclear |
| ***NIF vs. Control*** | | | | | | | |
| Amino, 2003 | High | High | High | Unclear | Unclear | Low | High |
| Yoshioka, 2006 | High | High | High | Unclear | Unclear | Unclear | High |
| ***AMD vs. NIF*** | | | | | | | |
| Amino, 2010 | High | High | High | Unclear | Unclear | Low | High |

Supplementary Table S3. The risk of bias assessment for non-randomized studies (RoBANS)

| Comparison | Selection of participants | Confounding variables | Measurement of exposure | Blinding of outcome assessments | Incomplete outcome data | Selective outcome reporting | Assessment of risk of bias across study |
| --- | --- | --- | --- | --- | --- | --- | --- |
|
| ***AMD vs. Control*** | | | | | | | |
| Piccini, 2011 | Low | Low | Low | Low | Low | Low | Low |
| Perzanowski, 2003 | High | Unclear | Unclear | Low | Unclear | High | High |
| Skrifvars, 2004 | Low | High | Low | Low | Low | Low | High |
| Pollak, 2005 | Low | High | Low | Low | Low | Low | High |
| Rea, 2006 | Low | High | Low | Low | Unclear | Low | High |
| Huang, 2015 | Unclear | Unclear | Low | Low | Unclear | High | High |
| Kubo, 2011 | Low | Unclear | Low | Low | Unclear | Low | Unclear |
| Watanabe, 2011 | Low | Unclear | Low | Low | Unclear | Low | Unclear |
| ***NIF vs. Control*** | | | | | | | |
| Shimizu, 2004 | Low | High | Low | Low | Low | Low | High |
| Ando, 2005 | High | Low | Low | Low | Low | Low | High |
| Igarashi, 2006 | Low | High | Low | Low | Unclear | Low | High |
| Tahara, 2006 | High | High | Low | Low | Low | Low | High |
| Shiga, 2010 | High | High | Low | Low | Low | Low | High |
| Igarashi, 2003 | Low | Unclear | Low | Low | Unclear | Low | Unclear |
| Tahara (2), 2010 | Low | Unclear | Low | Low | Unclear | Low | Unclear |
| Tahara (3), 2010 | Low | Unclear | Low | Low | Unclear | Low | Unclear |
| ***AMD vs. NIF*** | | | | | | | |
| Harayama, 2014 | Low | High | Low | Low | Low | Low | High |
| Yamazaki, 2010 | Low | Unclear | Low | Low | Unclear | Low | Unclear |
| Mera, 2010 | Low | Unclear | Low | Low | Unclear | Unclear | Unclear |
| Hayakawa, 2011 | Low | Unclear | Low | Low | Unclear | Unclear | Unclear |
| Yamamoto, 2012 | Low | Unclear | Low | Low | Unclear | Low | Unclear |
| Takenaka, 2013 | Low | Unclear | Low | Low | Unclear | Unclear | Unclear |
| Tagami, 2016 | Low | Low | Low | Low | Unclear | Low | Unclear |
| ***AMD vs. NIF vs. Control*** | | | | | | | |
| Amino, 2015 | Low | Low | Low | Low | Low | Low | Low |
| Tahara (1), 2010 | High | Unclear | Low | Low | Unclear | Low | High |
| Ito, 2010 | Low | Unclear | Low | Low | Unclear | Low | Unclear |

Abbreviation: AMD, amiodarone; NIF, nifekalant.

Supplementary Table S4. The Preferred Reporting Items for Systematic Reviews and Meta**-**Analyses (PRISMA**)** checklist

| **Section/topic** | **#** | **Checklist item** | **Reported on page #** |
| --- | --- | --- | --- |
| **TITLE** | | |  |
| Title | 1 | Identify the report as a systematic review, meta-analysis, or both. | 1 |
| **ABSTRACT** | | |  |
| Structured summary | 2 | Provide a structured summary including, as applicable: background; objectives; data sources; study eligibility criteria, participants, and interventions; study appraisal and synthesis methods; results; limitations; conclusions and implications of key findings; systematic review registration number. | 5 |
| **INTRODUCTION** | | |  |
| Rationale | 3 | Describe the rationale for the review in the context of what is already known. | 6 |
| Objectives | 4 | Provide an explicit statement of questions being addressed with reference to participants, interventions, comparisons, outcomes, and study design (PICOS). | 7 |
| **METHODS** | | |  |
| Protocol and registration | 5 | Indicate if a review protocol exists, if and where it can be accessed (e.g., Web address), and, if available, provide registration information including registration number. | NA |
| Eligibility criteria | 6 | Specify study characteristics (e.g., PICOS, length of follow-up) and report characteristics (e.g., years considered, language, publication status) used as criteria for eligibility, giving rationale. | 16-17 |
| Information sources | 7 | Describe all information sources (e.g., databases with dates of coverage, contact with study authors to identify additional studies) in the search and date last searched. | 17 |
| Search | 8 | Present full electronic search strategy for at least one database, including any limits used, such that it could be repeated. | 17 |
| Study selection | 9 | State the process for selecting studies (i.e., screening, eligibility, included in systematic review, and, if applicable, included in the meta-analysis). | 17 |
| Data collection process | 10 | Describe method of data extraction from reports (e.g., piloted forms, independently, in duplicate) and any processes for obtaining and confirming data from investigators. | 18 |
| Data items | 11 | List and define all variables for which data were sought (e.g., PICOS, funding sources) and any assumptions and simplifications made. | 16-17 |
| Risk of bias in individual studies | 12 | Describe methods used for assessing risk of bias of individual studies (including specification of whether this was done at the study or outcome level), and how this information is to be used in any data synthesis. | 18-19 |
| Summary measures | 13 | State the principal summary measures (e.g., risk ratio, difference in means). | 19 |

| Synthesis of results | 14 | Describe the methods of handling data and combining results of studies, if done, including measures of consistency (e.g., I^2^) for each meta-analysis. | 19 |
| --- | --- | --- | --- |
| Risk of bias across studies | 15 | Specify any assessment of risk of bias that may affect the cumulative evidence (e.g., publication bias, selective reporting within studies). | 20 |
| Additional analyses | 16 | Describe methods of additional analyses (e.g., sensitivity or subgroup analyses, meta-regression), if done, indicating which were pre-specified. | 20 |
| **RESULTS** | | |  |
| Study selection | 17 | Give numbers of studies screened, assessed for eligibility, and included in the review, with reasons for exclusions at each stage, ideally with a flow diagram. | 7 |
| Study characteristics | 18 | For each study, present characteristics for which data were extracted (e.g., study size, PICOS, follow-up period) and provide the citations. | 7 |
| Risk of bias within studies | 19 | Present data on risk of bias of each study and, if available, any outcome level assessment (see item 12). | 7-8 |
| Results of individual studies | 20 | For all outcomes considered (benefits or harms), present, for each study: (a) simple summary data for each intervention group (b) effect estimates and confidence intervals, ideally with a forest plot. | 8-9 |
| Synthesis of results | 21 | Present results of each meta-analysis done, including confidence intervals and measures of consistency. | 8-9 |
| Risk of bias across studies | 22 | Present results of any assessment of risk of bias across studies (see Item 15). | 7-9 |
| Additional analysis | 23 | Give results of additional analyses, if done (e.g., sensitivity or subgroup analyses, meta-regression [see Item 16]). | 9-10 |
| **DISCUSSION** | | |  |
| Summary of evidence | 24 | Summarize the main findings including the strength of evidence for each main outcome; consider their relevance to key groups (e.g., healthcare providers, users, and policy makers). | 11-15 |
| Limitations | 25 | Discuss limitations at study and outcome level (e.g., risk of bias), and at review-level (e.g., incomplete retrieval of identified research, reporting bias). | 15-16 |
| Conclusions | 26 | Provide a general interpretation of the results in the context of other evidence, and implications for future research. | 16 |
| **FUNDING** | | |  |
| Funding | 27 | Describe sources of funding for the systematic review and other support (e.g., supply of data); role of funders for the systematic review. | 3 |

*From:*  Moher D, Liberati A, Tetzlaff J, Altman DG, The PRISMA Group (2009). Preferred Reporting Items for Systematic Reviews and Meta-Analyses: The PRISMA Statement. PLoS Med 6(7): e1000097. doi:10.1371/journal.pmed1000097

For more information, visit: **www.prisma-statement.org**.

| Supplementary Table S5. Search strategies | | |  |
| --- | --- | --- | --- |
|  | **Database** | **Search terms** | **Results**  **found** |
|  |  |  |  |
|  | Pubmed | #1: Amiodarone [Mesh]: 7134 | 766 |
|  |  | #2: Nifekalant [Supplementary Concept]: 111 |  |
|  |  | #3: #1 OR #2: 7227 |  |
|  |  | #4: Cardiac arrest [TIAB]: 26399 |  |
|  |  | #5: Ventricular fibrillation [Mesh]: 15990 |  |
|  |  | #6: Pulseless ventricular tachycardia [TIAB]: 252 |  |
|  |  | #7: #4 OR #5 OR #6: 40475 |  |
|  |  | #8: #3 AND #7: 766 |  |
|  |  |  |  |
|  | Cochrane Central Register of Controlled Trails (CENTRAL) | #1: Amiodarone: ti,ab,kw: 1147 | 311 |
|  |  | #2: Nifekalant: ti,ab,kw: 10 |  |
|  |  | #3: Cardiac arrest: ti,ab,kw: 2362 |  |
|  |  | #4: Ventricular fibrillation: ti,ab,kw: 2430 |  |
|  |  | #5: Pulseless ventricular tachycardia: ti,ab,kw: 32 |  |
|  |  | #6: #1 AND #3: 46 |  |
|  |  | #7: #1 AND #4: 254 |  |
|  |  | #8: #1 AND #5: 6 |  |
|  |  | #9: #2 AND #3: 0 |  |
|  |  | #10: #2 AND #4: 5 |  |
|  |  | #11: #2 AND #5: 0 |  |
|  |  | #12: #6 OR #7 OR #8 OR #9 OR #10 OR #11:311 |  |
|  |  |  |  |
|  | Igaku Chuo Zasshi (ICHUSHI) | #1: Amiodarone/TH: 4245 | 970 |
|  |  | #2: Nifekalant/TH: 1027 |  |
|  |  | #3: #1 OR #2: 4889 |  |
|  |  | #4: Cardiac arrest/AL: 26257 |  |
|  |  | #5: Ventricular fibrillation/TH: 9227 |  |
|  |  | #6: Pulseless ventricular tachycardia/AL: 61 |  |
|  |  | #7: #4 OR #5 OR #6: 33383 |  |
|  |  | #8: #3 AND #7: 970 |  |
|  |  |  |  |
